# Cardiovascular markers of inflammation and serum lipid levels in HIV-infected patients with undetectable viraemia

**DOI:** 10.7448/IAS.17.4.19548

**Published:** 2014-11-02

**Authors:** Klaudija Viskovic, Snjezana Zidovec-Lepej, Lana Gorenec, Ivana Grgic, Davorka Lukas, Sime Zekan, Anja Dragobratovic, Josip Begovac

**Affiliations:** 1 Radiology and Ultrasound, University Hospital for Infectious Diseases, Zagreb, Croatia; 2 Department of Molecular Diagnostics and Flow Cytometry, University Hospital for Infectious Diseases, Zagreb, Croatia; 3 Reference Center for Diagnostics and Treatment of, Zagreb, University Hospital for Infectious Diseases, Croatia

## Abstract

**Introduction:**

Successfully treated HIV-infected patients may still have an increased risk for cardiovascular morbidity and mortality, which might be related not only to traditional risks, but also to inflammation and dyslipidemia induced by HIV and/or antiretroviral therapy [1, 2]. We examined the relationship of serum lipid levels with plasma biomarkers of inflammation using a composite inflammatory burden score (IBS) from the following seven markers of inflammation: CD40L, tPA, MCP-1, IL-8, IL-6, hCRP and P-selectin.

**Materials and Methods:**

Subjects were selected among consecutive HIV-infected males ≥18 years of age with an undetectable viral load (<50 copies/mL of HIV1-RNA), seen at the University Hospital for Infectious Diseases, Zagreb, Croatia, in the period from January 2012 to March 2013. Plasma inflammatory biomarkers (CD40L, tPA, MCP-1, IL-8, IL-6, hCRP and P-selectin, quantified by bead-based cytometry) >75th percentile were considered elevated and an IBS was constructed as the presence of zero, one, two, or three or more elevated biomarkers. Correlations between the IBS and lipid parameters were examined using Spearman's Rho and by ordered logistic regression proportional odds model to estimate the odds of more elevated (>75th percentile) biomarkers.

**Results:**

181 male patients were included into the study, the median age was 46.7 (Q1–Q3, 39.9–55.0) years and the median current CD4 cell count was 553.0 (Q1–Q3, 389–729) per microliter. The patients were mainly treated with two nucleoside reverse transcriptase inhibitor (NRTI) plus one non-NRTI (NNRTI) (N=100, 60.8%) or two NRTI plus lopinavir (N=50, 27.6%). There was a significant correlation between the IBS and serum cholesterol (Rho=0.23, 95% CI, 0.09–0.37), triglycerides (Rho=0.30, 95% CI, 0.16–0.42) and cholesterol/HDL-cholesterol ratio (Rho=0.25, 95% CI 0.11–0.38). In the multivariable model a one unit increase in cholesterol/HDL-cholesterol ratio was associated with a 1.72-fold (95% CI, 1.27–2.33) increased odds of having a greater IBS. One unit increase (mmol/L) of cholesterol and triglycerides was associated with a 1.41-fold (95% CI, 1.13–1.76) and 1.37-fold (95% CI, 1.18–1.60) increased odds of having a greater IBS, respectively.

**Conclusions:**

Our study suggests that in virologically suppressed patients there is a significant association between markers of inflammation and serum levels of cholesterol and triglycerides as well as the cholesterol/HDL-cholesterol ratio.
